# Cell and tissue system capable of automated culture, stimulation, and monitor with the aim of feedback control of organs-on-a-chip

**DOI:** 10.1038/s41598-020-80447-2

**Published:** 2021-02-04

**Authors:** Satoshi Konishi, Takeshi Hashimoto, Tsubasa Nakabuchi, Takatoshi Ozeki, Hiroki Kajita

**Affiliations:** 1 Department of Mechanical Engineering, College of Science and Engineering, Ritsumeikan University, Kusatsu, 525-8577 Japan; 2 Graduate Course of Science and Engineering, Ritsumeikan University, Kusatsu, 525-8577 Japan; 3 Ritsumeikan Global Innovation Research Organization, Ritsumeikan University, Kusatsu, 525-8577 Japan; 4 College of Sport and Health Science, Ritsumeikan University, Kusatsu, 525-8577 Japan

**Keywords:** Assay systems, Tissue engineering, Data processing

## Abstract

This paper presents progress in the automation of cell and tissue systems and attempts toward the in situ feedback control of organs-on-a-chip. Our study aims to achieve feedback control of a cell and tissue system by a personal computer (PC), whereas most studies on organs-on-a-chip focus on the automation of status monitoring. The implemented system is composed of subsystems including automated culture, stimulation, and monitoring. The monitoring function provides imaging as well as sampling and dispensing in combination with an external analyzer. Individual subsystems can be combined accordingly. First, monitoring of skeletal muscle (SM) and adipose tissues using this system was demonstrated. The highlight of this paper is the application of the system to the feedback control of the lipid droplet (LD) size, where biochemical stimulation using insulin and adrenaline is controlled by a PC according to the obtained LD imaging data. In this study, the system demonstrated its function of maintaining the desired size of LDs. Our results expand the possibility of PC-controllable cell and tissue systems by addressing the challenge of feedback control of organs-on-a-chip. The PC-controllable cell and tissue systems will contribute to living systems-on-a-chip based on homeostasis phenomena involving interactions between organs or tissues.

## Introduction

Tissue engineering using cultured cells has promising potential in the fields of regenerative medicine and drug discovery in addition to its potential in the field of pure life science. The integration of detectors and functional fluidic microdevices has allowed the development of miniaturized biochemical reactors and analyzers^1^. The lab-on-a-chip has been widely studied in micro total analysis systems and has been applied to cell culture-on-a-chip^2^. Neurons-on-a-chip with microelectrode arrays have been studied for a long time to investigate their physiological activity^3–5^. Recently, various cells-on-a-chip have been cultured and investigated, and even three-dimensional tissues-on-a-chip are commonly used for research in the field. Many studies on organs-on-a-chip have been reported over the last decade^6^. Cellular tissue-on-a-chip systems may contribute to drug screening as a powerful alternative to conventional animal experiments. Reported organs-on-a-chip include the heart-on-a-chip^7^, lung-on-a-chip^8^, gut-on-a-chip^9^, and an artificial intestinal tract system^10^. In addition, various interactions between different organs-on-a-chip have been studied^11–15^, and research on body-on-a-chip systems^16–18^ has become popular. For example, a two-organ microphysiological system composed of intestinal Caco-2 and hepatic HepaRG cells^12^ and even a four-organ (cardiac, liver, SM and neuron) system^18^ have been reported.

In the fields of tissue engineering, various automated cell culture systems have been developed, and some have been commercialized to improve the production efficacy of cells^19–21^. Among these systems, an automated cell culture system for human induced pluripotent stem cells demonstrated attractive performance metrics^19^, including automated cell seeding, medium changing, cell imaging, and cell harvesting.

We are interested in SM^22–24^ and adipose tissue^25^. An in vitro engineered SM has been studied as an alternative to animal models for disease modeling and drug discovery related to human muscle diseases^26–30^. SM produces various metabolites (e.g., lactate) during exercise. Changes in metabolism when SM is electrically stimulated are analyzed by continual automated sampling, which enables monitoring of the status of exercising SM, and the feedback data will contribute to the future assessment of external stimuli. Adipose tissue is sensitive to insulin^31^. Excessive lipid accumulation in adipose tissue/adipocytes is a central feature of obesity and metabolic syndrome. LDs, in which excess energy is stored as triglycerides (TGs), are considered as active organelles involved in diverse cellular processes, such as membrane trafficking and lipid metabolism^25,32^. Moreover, crosstalk between SM and other tissues and organs has been studied by other groups to establish organs-on-a-chip as more realistic in vitro model^10–18^. Among these biological materials, adipose tissue is important in terms of the crosstalk with SM. The size of LDs is affected by exercise^33^. One study of a coculture of SM and adipose tissue investigated adipocytes and muscle metabolism^34^. SM and adipose tissues are suitable for the application in a total system combining automated functions, including culture, electrical stimulation, biochemical stimulation, imaging, sampling and dispensing, and linked to an analyzer.

With respect to organs-on-a-chip for SM, a muscle-on-a-chip with on-site multiplexed biosensing was reported by another group^23^. The reported muscles-on-a-chip have aimed at in situ monitoring of biomarkers and performed multiple-time-point measurements. It has been reported that integrated biosensors enable in situ monitoring. In general, integrated biosensors can provide the advantages of a compact size and fast response, which could be applied to our feedback system if necessary. On the other hand, an external analyzer can provide the best desired performance without restrictions due to the integration in a limited space. This study designs and employs biosensing combined with an external analyzer, providing precision and multiple sensing to explore further possibilities. Functions including continual extraction and dispensing of samples for metabolic analyses are designed and implemented. Samples, which are separately stored in containers, are transported to the external analyzer. The combination of automated sampling with an external analyzer is applied to the SM system to evaluate the reaction of cellular tissue to PC-controlled electrical stimulation. This study aims to achieve the feedback control of the response of cultured cells, whereas most of the reported organs-on-a-chip were designed for the in situ monitoring of cellular tissue^11,35^. The PC-controllable cell and tissue system enables feedback control in addition to the function of in situ monitoring. This paper presents feedback control of size changes in LDs, where a desired size of LDs is maintained.

## Results and discussion

### PC-controllable cell and tissue system

**Figure 1:**
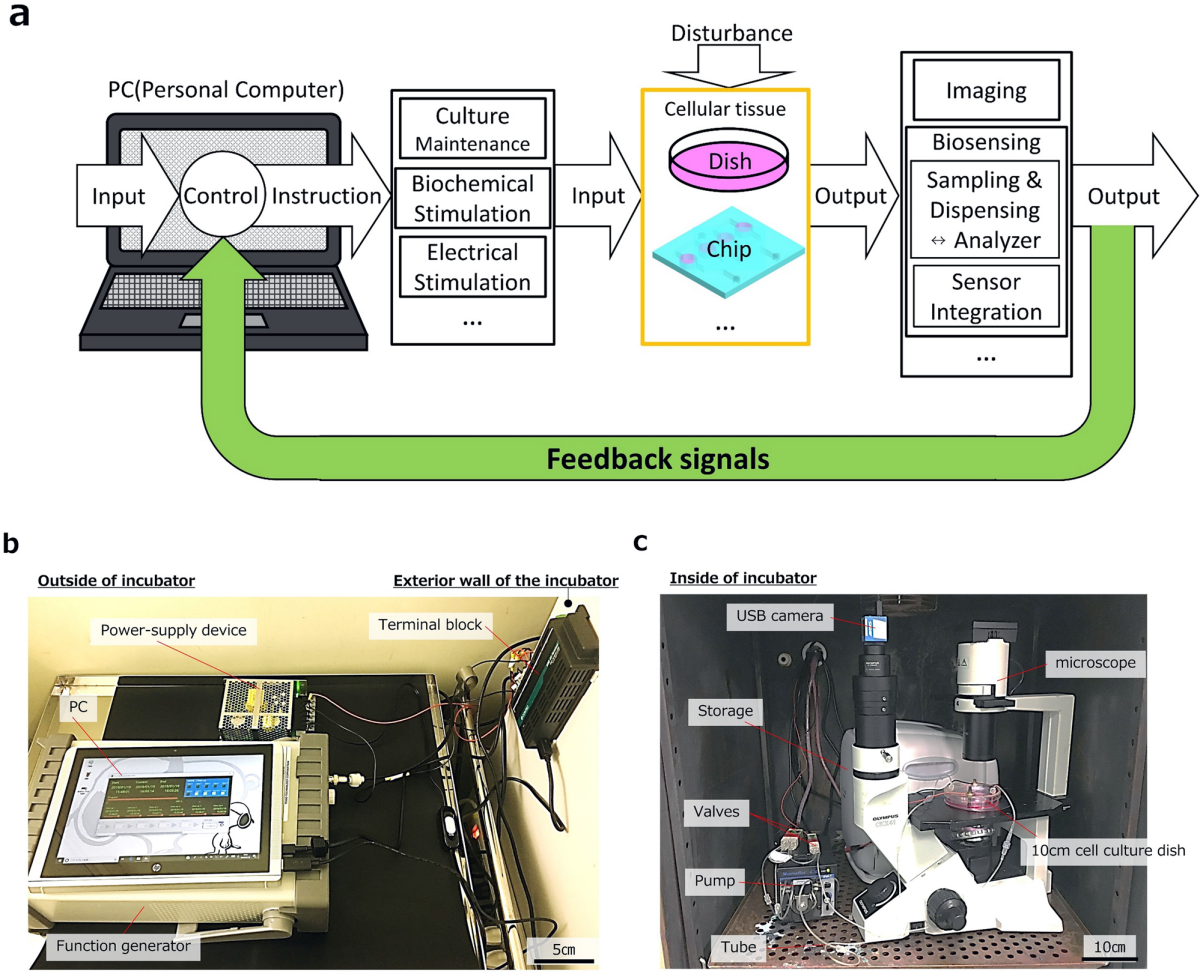
Conception and composition of a PC-controllable cell and tissue system. (**a**) Schematic image of a system composed of subsystems for feedback control. The system is composed of subsystems in charge of automaton of culture media exchange, electrical or biochemical stimulation, imaging, sampling and dispensing. (**b**) Photograph of PC and peripheral electronics outside an incubator. PC and a function generator were connected to equipment in an incubator via a terminal block on an exterior wall of the incubator. The terminal block with a power supply device was shown at the right-hand side of the photograph. (**c**) Photograph of fluidic systems, a microscope, and a camera in the incubator. A 10 cm dish for culture of cellular tissue was placed on a stage of the microscope and was equipped with fluidic systems of a pump and valves in the incubator. The equipment outside/inside the incubator were connected by the terminal block and tubing through the wall of the incubator.

This study designs and develops a PC-controllable cell and tissue system, as shown in Fig. 1a. The system is composed of subsystems in charge of automation of culture media exchange, electrical or biochemical stimulation, and monitoring. The monitoring function includes imaging, sampling and dispensing. Samples are analyzed in combination with an external analyzer as explained. Photographs of implemented subsystems corresponding to Fig. 1a are shown in Fig. 1b,c. PC and peripheral electronics were set outside an incubator for culture of cellular tissue as shown in Fig. 1b. Figure 1c shows fluidic systems and a microscope with a camera in the incubator. The equipment outside/inside the incubator were connected by the terminal block and tubing through the wall of the incubator. The demonstrated system is characterized by continual sampling and dispensing by combination with an external precise analyzer. The system performance using SM is demonstrated and compared with that of conventional sampling by manual pipetting. SM produces various metabolites during exercise. The changes in metabolism when SM is electrically stimulated are analyzed. In addition, the system is characterized by automated imaging by a microscope installed in the incubator and simultaneous transmission of the images to a PC for visual feedback control. Images transmitted to the PC are processed to analyze the behavior, such as motion and shape changes, of cellular tissues. Contractions of SM and size changes in adipocyte LDs are evaluated by automated imaging. Finally, feedback control of the size of LDs by controlling the addition of biochemical stimulants is demonstrated to verify the system performance.

**Figure 2:**
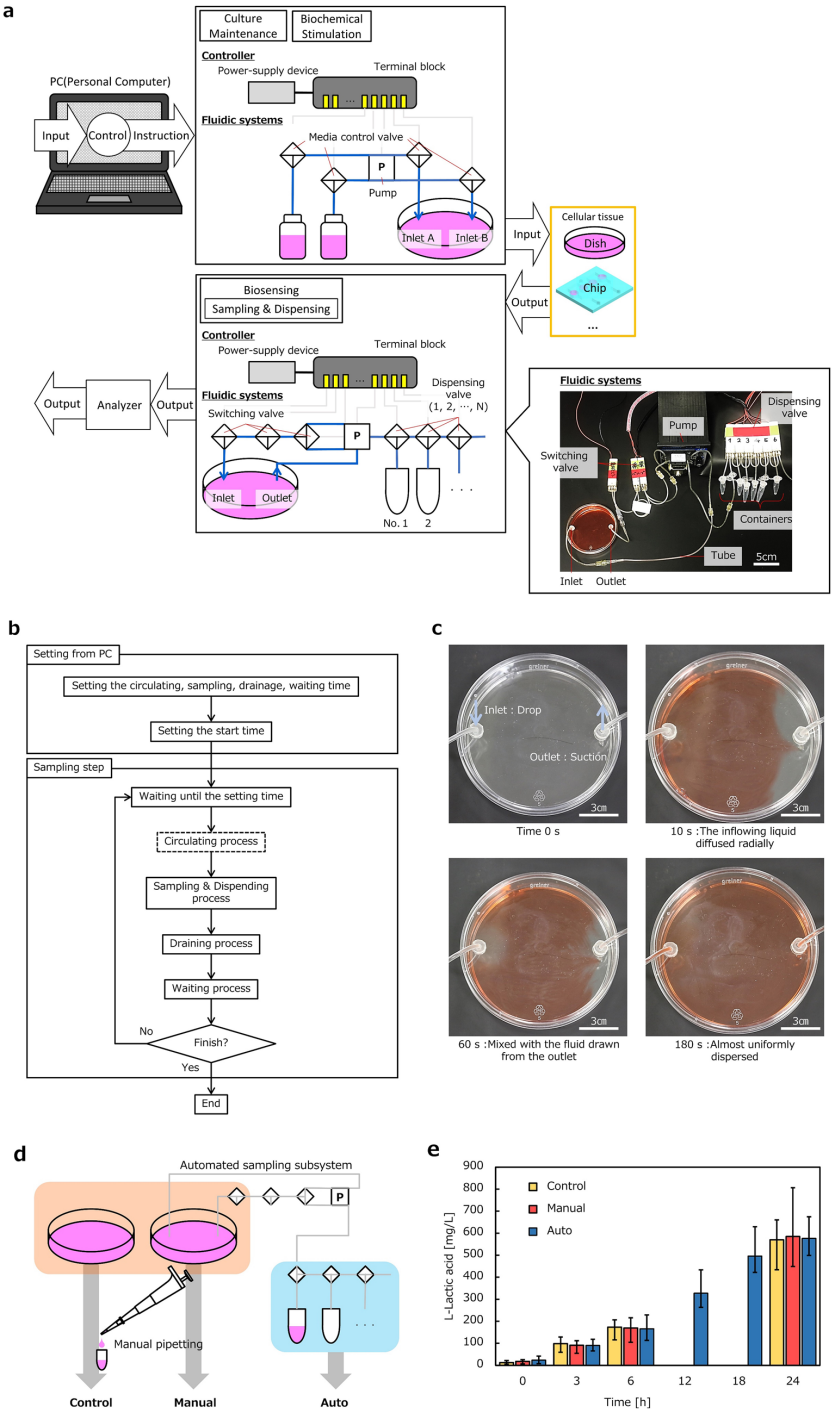
Culture subsystem and sampling and dispensing subsystem. (**a**) Subsystem composition: PC, Block of culture maintenance and biochemical stimulation, Culture environment for cellular tissue such as dish and chip, Biosensing block consisting subsystem of sampling and dispensing with an analyzer. A terminal block connected the PC with electrical equipment for subsystems. Photograph of fluidic systems of a pump and valves in the incubator are also presented. The fluidic systems were connected to a 10 cm dish for culture of cellular tissue. (**b**) Flow chart of an algorism for PC-control of sampling. Sampling operation progresses in four steps: Circulating, Sampling, Drainage, Waiting. (**c**) Sequential photographs of liquid distribution in a culture dish, which was uniformized by the circulating step. The red-colored liquid was uniformly distributed. (**d**) Measurement of l-lactic acid as a metabolite from the SM cellular tissue. In the evaluation, sampling performance of an automated sampling subsystem were compared with sampling by manual pipetting as control. (**e**) Measurement result of l-lactic acid as a metabolite from the SM cellular tissue (n = 3 in each condition). l-lactic acid increased in the same way for both automated and manual sampling. Note that automated sampling was available at 12 and 18 h.

**Figure 3:**
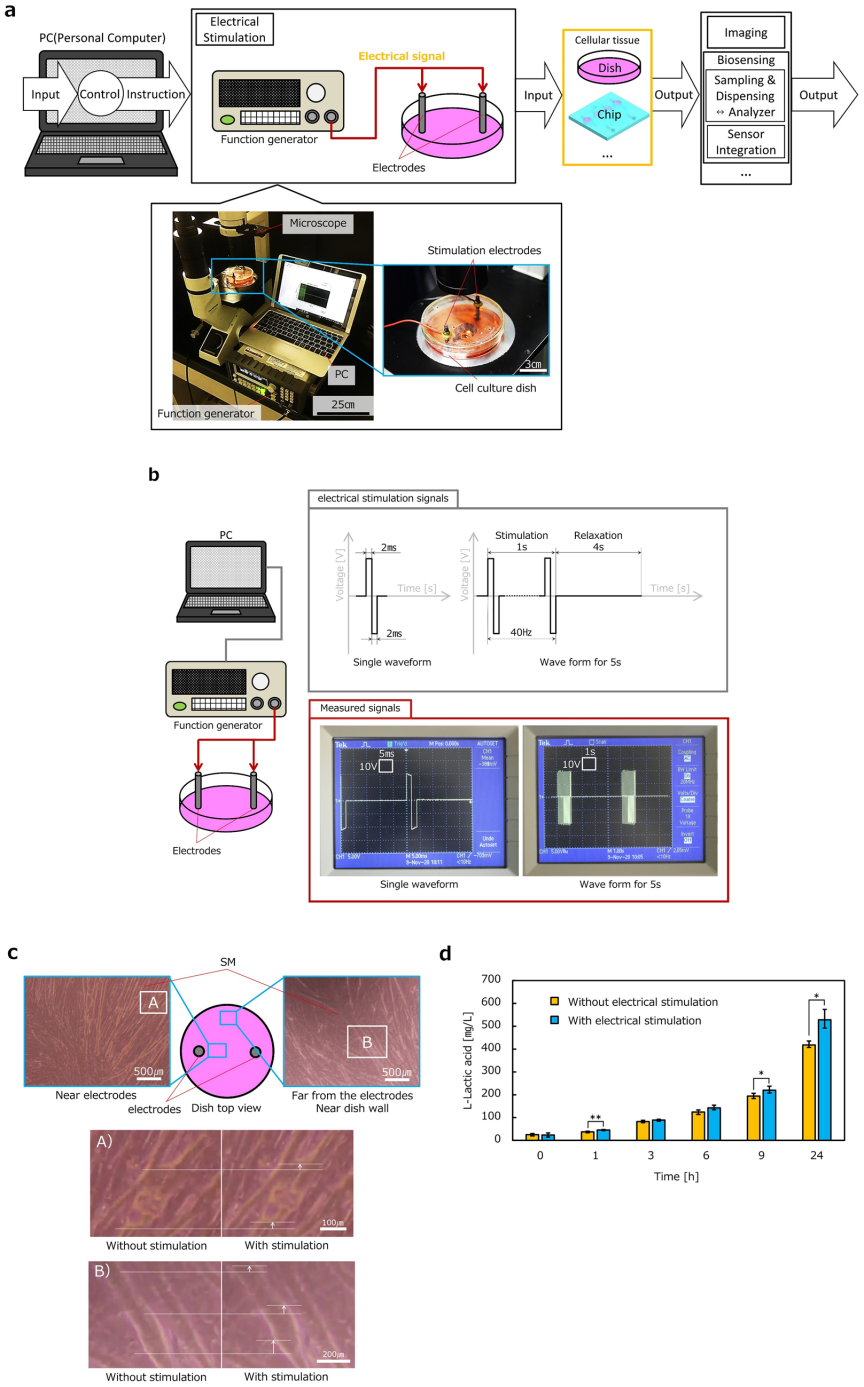
Electrical stimulation subsystem. (**a**) Subsystem composition: PC, Electrical stimulation block, Culture environment for cellular tissue, Biosensing block, Imaging block. Photographs of implemented electrical stimulation subsystem was also presented. A cell culture dish on a stage of microscope was equipped with stimulation electrodes. (**b**) Single waveform together with waveform for 5 s. Pictures of real signals captured from display of an oscilloscope and corresponding drawing were shown. Rectangular waves were 40 signals of 4 ms for electrical stimulation were applied in 1 s with the aim of inducing maximum contraction due to complete tetanus. Generated outputs according to PC instruction and measured signals were captured as shown in the lower part of (**b**). The time and the voltage were taken on the lateral and vertical axes, respectively and voltage. Scales of each axis were 10 V and 500 ms. 40 rectangular waves were generated in 1 s as the stimulation signal. The stimulation signal was repeatedly measured every 4 s relaxation. (**c**) Evaluation of SM cellular tissue contraction induced by electrical stimulation. A 10 cm dish was prepared for culture of SM cellular tissue and electrodes were set at distance of 5 cm. Imaging subsystem was used to observe SM cellular tissue. Two regions of SM cellular tissue cultured in a dish were extracted and magnified. Images without and with the stimulation were compared for both of two regions in upper part of (**c**). The contraction of SM was confirmed at both regions. (**d**) Measurement of l-lactic acid as a metabolite from the SM cellular tissue (n = 3 in each condition). The time course change in l-lactic acid concentration showed interaction between condition and time (*p* < 0.001). Electrical stimulation caused higher concentration of l-lactic acid after 1, 9, and 24 h than control (*p < 0.05, **p < 0.01).

**Figure 4:**
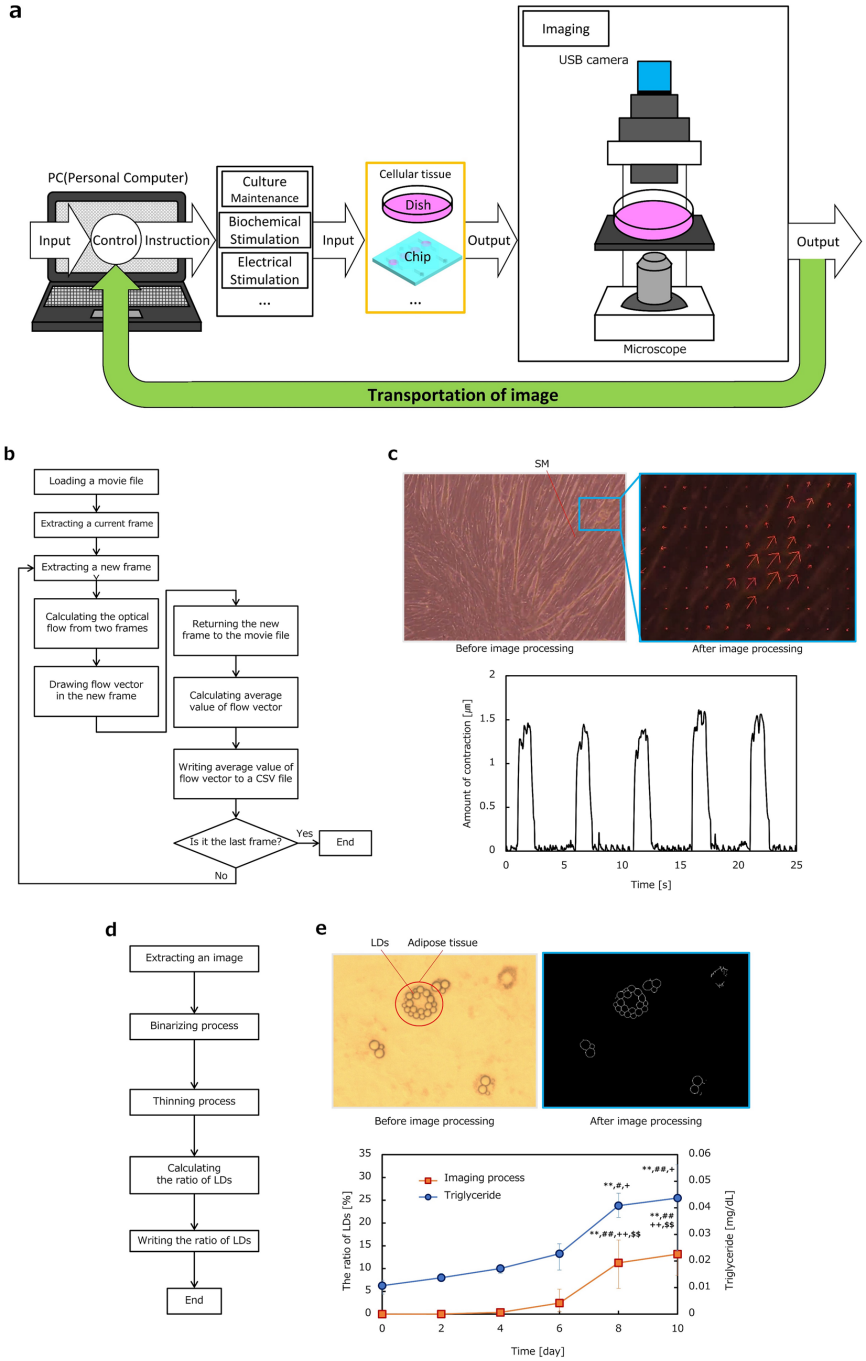
Imaging subsystem. (**a**) Subsystem composition: PC, Electrical and biochemical stimulation block, Culture environment for cellular tissue, Imaging block. Images are transmitted to PC and processed by program in PC to evaluate status of cellular tissue and the evaluation result is utilized for feedback instruction to the system. (**b**) Flow chart of an algorism for PC-controllable imaging of SM cellular tissue contraction. (**c**) Imaging and Image processing of the SM cellular tissue contraction. Optical flow based on the difference between frames was shown in a picture after image processing. Estimated contraction displacement was presented in a graph of (**c**). (**d**) Flow chart of an algorism for PC-controllable imaging of lipid droplets (LDs) of adipose cellular tissue. (**e**) Imaging and Image processing of size change of LDs. Binarized and thinned image of LDs was shown in a picture after image processing. Estimated change in the ratio of white pixels as a correlation index to the LD size according to time was presented in a graph of (**e**) (n = 10). In the graph, triglyceride in adipose cells were also biochemically assessed (n = 3, three independent experiments in each time point). Both the ratio and triglyceride concentration increased in similar way. **p < 0.01 vs. day 0; ^#^*p* < 0.05, ^##^*p* < 0.01 vs. day 2; ^+^*p* < 0.05, ^++^*p* < 0.01 vs. day 4; ^$$^*p* < 0.01 vs. day 6.

The PC-controllable cell and tissue system was implemented as shown in Fig. 1b,c. The implemented system consisted of elemental subsystems: a culture subsystem together with a sampling and dispensing subsystem (Fig. 2), an electrical stimulation subsystem (Fig. 3), and an imaging subsystem (Fig. 4). The details of individual subsystems will be described below.

### Culture subsystem and sampling and dispensing subsystem

First, the subsystems for culture and for sampling and dispensing are described together because they share a common fluidic control system. The culture subsystem performs culture media exchange and biochemical stimulation by supplying liquid solution to the culture environment. The sampling and dispensing subsystem extracts samples from the culture subsystem and dispenses the extracted samples into separate containers such as a storage tip. Figure 2a shows a composition and corresponding pictures of implemented culture subsystem and sampling and dispensing subsystem. The subsystems for culture and for sampling and dispensing consist of a PC with peripheral electronics and fluidic systems. Culture media sampling is executed by a sequential operation of three-way valve and pump, which are controlled by the PC. A flow chart of the sequential steps for sampling operation is shown in Fig. 2b. Extracted samples are dispensed into storage containers separately. The number of containers is flexible depending on analytical requirements. The sampling operation proceeds via four steps: circulating, sampling, drainage, and waiting, as shown in Fig. 2b. The liquid solutions, such as culture media, is stirred by circulating flow in the circulating step. Liquid is transported in a tube, separated and preserved in the sampling step. The stored sample liquid volume is determined from the volume of the segment of the tube between the circulating valves. The remaining liquid in the tube is returned into the culture environment in the drainage step. Finally, the valves and pump are switched off in the waiting step. The frequency for sampling can be freely programmed even across the limitation of human operation due to PC-controlled automation.

Figure 2c shows sequential photographs of the liquid distribution in the culture environment, which is made uniform in the circulating step. Uniform conditions are important for culture media exchange as well as biochemical stimulation. As shown in Fig. 2c, with circulation-driven homogenization, red-colored liquid was introduced at the Inlet, and liquid from the culture environment was removed at the outlet. The red-colored liquid was distributed uniformly after 180 s by the circulating step. Figure 2c–e verifies the performance of the subsystems for supplying the liquid solution, sampling, and dispensing. SM cells were cultured in a dish, and the concentration changes in l-lactic acid, a typical metabolite produced by contracting SM, in the culture dish were measured. In the evaluation, the sampling performance of the automated sampling subsystem was compared with that of sampling by manual pipetting as a control (see the illustration in Fig. 2d). Manual pipetting from a dish equipped with the sampling subsystem was also evaluated. The graph in Fig. 2e shows the evaluated l-lactic acid concentrations in the culture environment. Sampling was executed at 0 h, 3 h, 6 h, 12 h, 18 h, and 24 h, where the time for sampling could be flexibly determined by setting in program. The l-lactic acid concentration increased in proportion to time along almost the same line for all three methods: the time course change in l-lactic acid concentration indicated a main effect of time (*p* < 0.001), but there was no main effect or interaction between the condition. Thus, the performance of the presented subsystems was verified.

### Electrical stimulation subsystem

Next, the subsystem for electrical stimulation is described as shown in Fig. 3a. Figure 3a shows the composition and corresponding pictures of the electrical stimulation subsystem. As shown in Fig. 3a, a dish was used as the culture environment, and two electrodes were set in the dish. As explained in Fig. 3a, a signal pattern for electrical stimulation was created by the PC as shown in the upper part of Fig. 3b and transmitted to a function generator. The function generator generates an electrical signal for stimulation based on the PC instructions and applies the generated signal through the electrodes. Electrical stimulation was mainly used for SM contraction in our study. SM contraction was observed and evaluated by the imaging subsystem. Figure 3b presents the signal pattern created by the PC and the corresponding electrical signal generated by the function generator. The electrical signal based on the PC instructions was measured through carbon rod electrodes by an oscilloscope. 40 signals of 4 ms for electrical stimulation were applied in 1 s with the aim of inducing maximum contraction due to complete tetanus. Generated outputs according to PC instruction and measured signals were captured as shown in the lower part of Fig. 3b. 40 rectangular waves in 1 s were repeatedly measured every 4 s of relaxation time. We can see the good agreement between generated and measured signals.

Figure 3c verifies the performance of the electrical stimulation subsystem. The magnitude of contraction was quantified using the image processing^36^. Two regions of SM cellular tissue cultured in a dish were extracted and are magnified in Fig. 3c. Images without and with stimulation are shown. SM contraction was confirmed in both regions (see supplemental Video S1). Moreover, l-lactic acid concentration in the dish was measured to evaluate the influence of electrical stimulation on metabolism. Figure 3d compares the measured l-lactic acid concentrations with and without electrical stimulation with respect to time. The l-lactic acid concentration increased with time in both cases (*p* < 0.001). SM with electrical stimulation showed an l-lactic acid concentration approximately 14% higher than that of the control after 6 h (*p* < 0.05). The difference reached approximately 26% after 24 h (*p* < 0.05). These results verify the performance of the electrical stimulation subsystem.

### Imaging subsystem

Next, the imaging subsystem is presented in Fig. 4a. The composition of the imaging subsystem is shown in Fig. 4a. Imaging is useful for monitoring culture conditions and evaluating cellular tissue behavior, such as SM contraction and size changes in adipocyte LDs. An inverted microscope was placed in the incubator for in situ observation in this study. Dynamically captured images were transmitted to the PC and processed by the program in the PC to evaluate the status of cellular tissue, and the evaluation results were utilized to provide feedback instructions to the system. SM contraction and size changes in adipocyte LDs were evaluated to verify the imaging subsystem performance.

Figure 4b provides a flow chart of the evaluation process based on dynamic images of SM cellular tissue in the culture dish. Dynamic images captured by the microscope were processed one frame at a time. The optical flow based on the difference between frames was used to evaluate the displacement of the contracting motion of SM cellular tissue, as shown in the image in Fig. 4c. The average value of the flow vector was calculated and used as the representative displacement, as explained in the flow chart in Fig. 4b. The magnitude of contraction was quantified using the image processing (see supplemental Video S2). The contracting displacement of the SM cellular tissue was obtained as shown in the graph in Fig. 4c. The conditions of electrical stimulation were set at 40 V_pp_ and 40 Hz. The stimulation duration time and the relaxation time were set at 1 s and 4 s, respectively.

Next, adipose cellular tissue was evaluated by the imaging subsystem. The Imaging algorism is explained in the flow chart in Fig. 4d. Imaging process using OpenCV (Intel Co.) was applied to captured photographs of adipocytes tissue with lipid droplets to calculate area of LDs in the image. The imaging process includes binarizing process and thinning process of the original images to recognize and extract LDs in the images. Captured images of LDs were processed by binarizing and thinning, as shown in the series of images in Fig. 4e. The ratio between the number of black pixels and white pixels after image processing was used as a correlation index to evaluate the size change in LDs, where the number of white pixels corresponded to the sum of circumference of LDs. Performance of developed subsystem was verified by preliminary experiment. The insulin-/adrenaline-dependent LDs formation/degradation has been reported, respectively^25,37^. Insulin as an accelerator was supplied until 12 h and then adrenaline as a decelerator was supplied after 12 h. It was found that the ratio of the correlation index to the LD size increased when the accelerator was supplied but decreased with the addition of the decelerator in our experiment. A graph of Fig. 4e shows the change in the ratio with time. The ratio to evaluate size change of LDs increased according to time as shown in Fig. 4e. TG in adipocytes were also biochemically assessed in the experiment, which is associated with the change of size change of LDs. TG also increased in similar way to the ratio as a correlation index to the LD size. We can see that the image processing is effective for evaluation of the size change of LDs as well as TG storage.

### Total system in combination with subsystems

Subsystems for culture, stimulation, imaging, sampling and dispensing were combined to demonstrate the total system performance. It was confirmed that the individual subsystems of the PC-controllable cell and tissue system performed successfully as designed. SM cellular tissue and adipose cellular tissue were used to verify the performance of each automated subsystem. Next, we will examine the total system performance with the combined subsystems. Further attempts toward feedback control of organs-on-a-chip will be reported and discussed.

### Total system performance with SM cellular tissue

**Figure 5:**
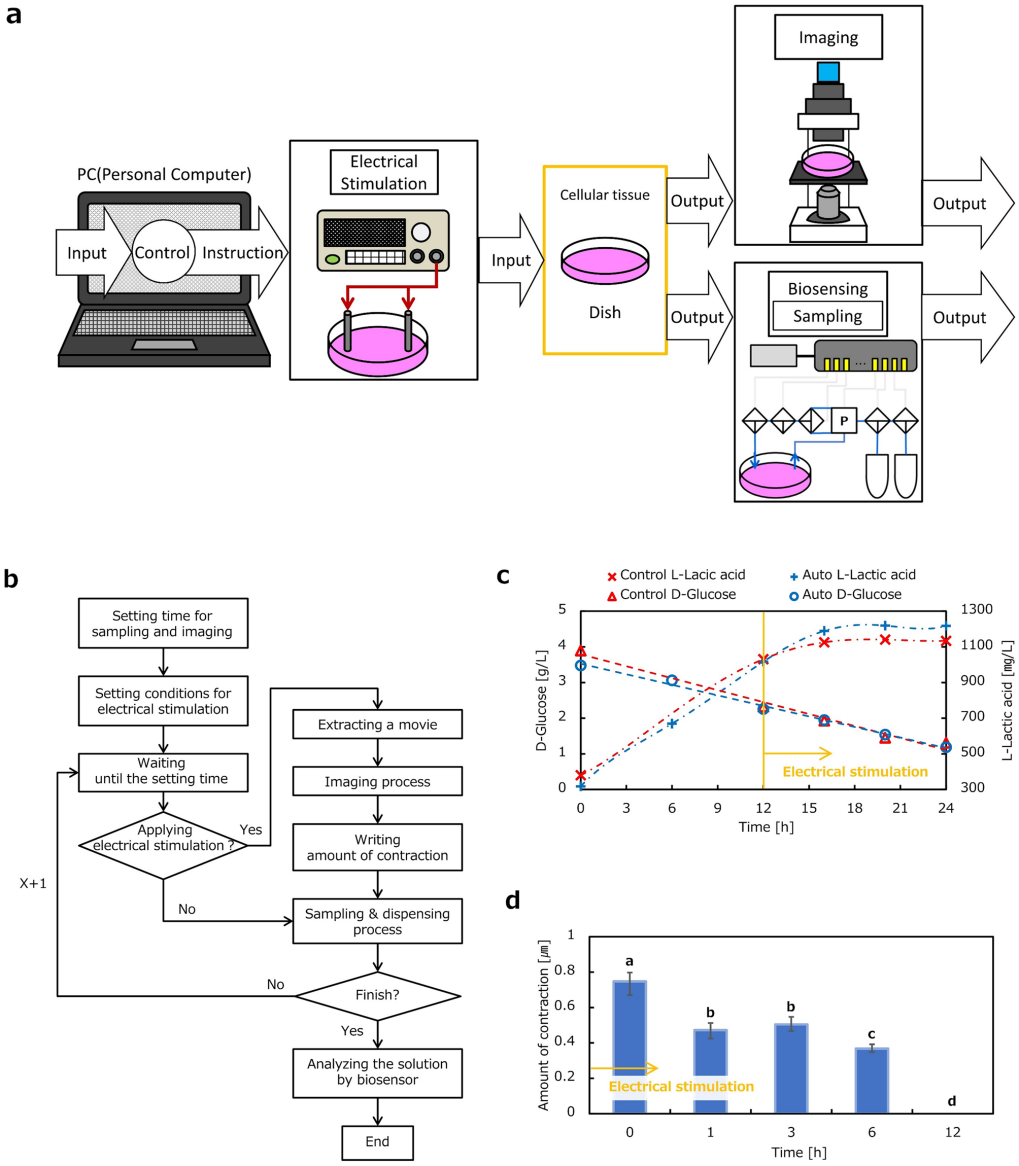
PC-controllable sampling, dispensing, analyzing of metabolite from the electrically stimulated SM cellular tissue together with imaging of its contraction. (**a**) A total system composition consisting of subsystems for culture, sampling and dispensing with an analyzer, electrical stimulation, and imaging. SM cellular tissue was electrically stimulated and evaluated by biosensing and imaging. The experiment was carried out for 24 h and electrical stimulation was applied after 12 h. (**b**) Flow chart of an algorism for PC-controllable imaging of SM cellular tissue contraction. (**c**) Measurement of d-Glucose in a culture media and l-lactic acid as a metabolite (n = 1). Electrical stimulation was likely to affect the concentration of l-lactic. (**d**) Evaluation of contraction of the SM cellular tissue by image processing. The average value of the flow vector was calculated as contraction of SM (n = 5). Contraction of SM cellular tissue showed stable value for several hours and decreased. Plots not connected by the same letter are significantly different from each other (one way ANOVA with Bonferroni comparisons, p < 0.01).

The automated sampling of metabolites from electrically stimulated SM cellular tissue was evaluated by combination with an external analyzer. Subsystems for culture, sampling and dispensing, electrical stimulation, and imaging were combined in the experiment as shown in Fig. 5a. SM cells were cultured in a dish equipped with the subsystems and the total system was placed in an incubator. Figure 5b provides a flow chart of the evaluation process based on dynamic images of SM cellular tissue in the culture dish.

Figures 5c,d show the experimentally measured values in the culture dish and contracting displacement during electrical stimulation. Figure 5c shows the changes in concentrations of d-glucose and l-lactic acid, which are both important metabolites, over time. A dish manually prepared in a conventional way was prepared as a control experiment and compared with a dish equipped with an automated total system. Electrical stimulation was applied from 12 to 24 h. The conditions were 40 V_pp_ and 40 Hz with 1 s and 4 s as the duration time and the relaxation time, respectively. The measurement results for both d-glucose and l-lactic acid in the dish equipped with automated subsystems showed good agreement with the results of the control experiment before electrical stimulation. The concentration of l-lactic acid increased to approximately 7% higher than that of the control due to electrical stimulation. The concentration of d-glucose was in good agreement with that of the control experiment after stimulation. The imaging subsystem automatically monitored the contracting displacement for 12 h during electrical stimulation. Images were captured for 25 s in each round of imaging.

Figure 5d shows average displacement of contraction in the captured image after 12 h. The results showed that the contraction of SM cellular tissue kept the stable value for several hours and decreased with time. We confirmed the cell viability by microscopic images and observed that cells were still intact in response to electrical stimulation (see and compare supplemental Figs. S1-(1) and (2)). This paper aims to demonstrate and verify the total system performance. The results show that the system achieved this aim; moreover, the obtained results were interesting for further experimental investigations using the system.

### Application of the total system to provide feedback control of adipose cellular tissue

**Figure 6:**
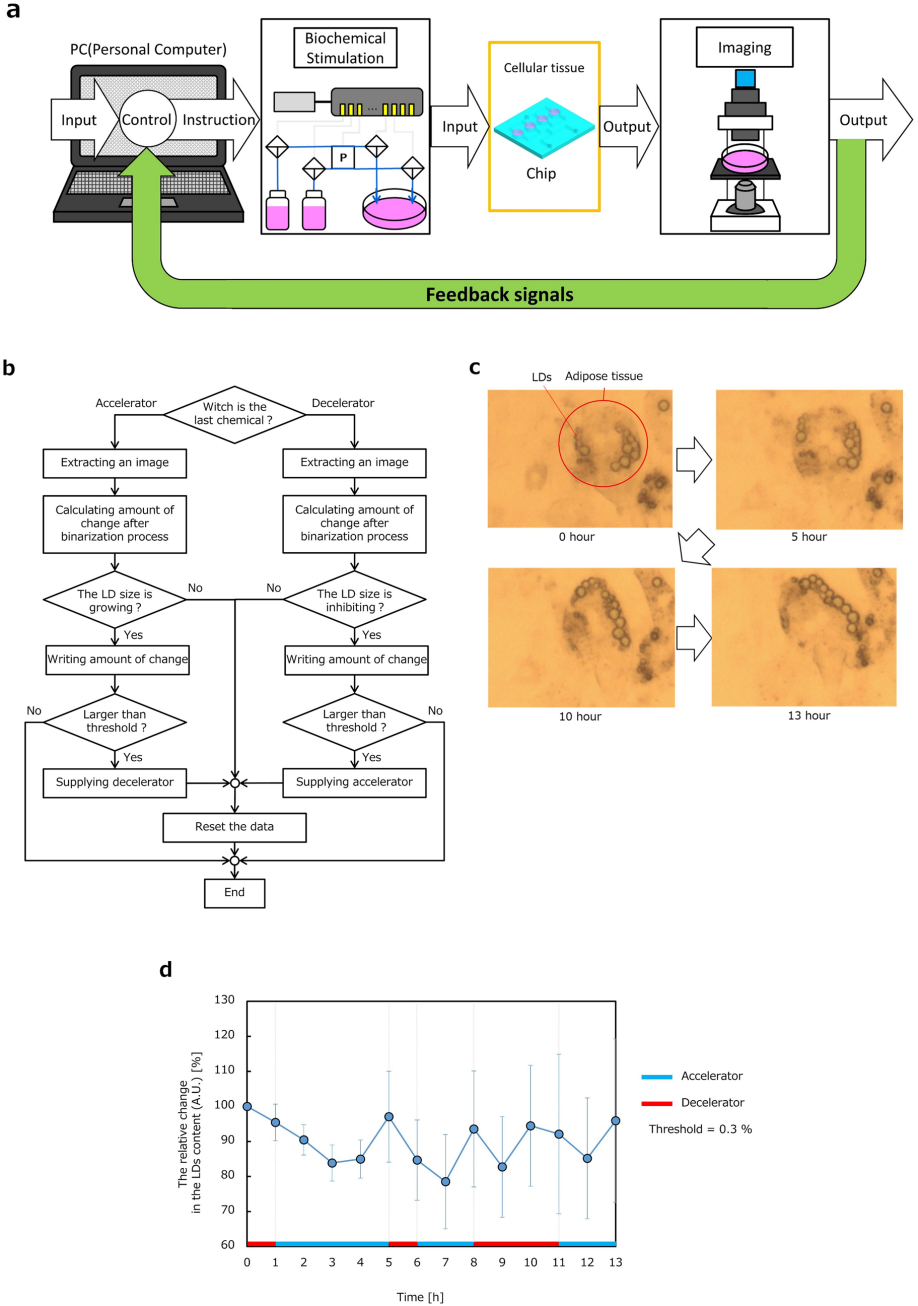
PC-controllable system for feedback control of LDs of adipose cellular tissue. (**a**) A total system composition consisting of subsystems for culture, biochemical stimulation, and imaging. Size change of LDs of adipose cellular tissue was monitored when biochemical stimulation using accelerator and decelerator. (**b**) Flow chart of an algorism for feedback control of LD size by controlling biochemical stimulation. (**c**) Photographs of continual images of adipose tissue and LDs according to time. Typical adipose tissue with LDs was indicated by using a red circle in (**c**). Cluster of LDs distributed and changed its shape and location in a frame. (**d**) The relative change in the LDs content obtained through the image processing by binarizing and thinning according to time (n = 6). The biochemical stimulation was automatically switched when the ratio of LDs changed over a set value as a threshold. The threshold was set at 0.3% of change in the ratio in the experiment. The feedback control of the size of LDs was successfully performed. The biochemical decelerator was automatically supplied when the ratio of LDs increased over a set value as a threshold, whereas accelerator was automatically supplied when LDs decreased over the threshold.

Finally, feedback control using our system is reported. The implemented PC-controllable cell and tissue system was applied to provide feedback control of the size of LDs of adipose cellular tissue. Biochemical stimulation and image processing were combined to control the size of LDs. Subsystems for culture, sampling and dispensing, biochemical stimulation, and imaging were combined in the experiment, as shown in Fig. 6a. Biochemical stimulation according to PC instructions based on images of LDs was used to control and stabilize changes in the size of LDs. The algorism is explained by the flow chart in Fig. 6b. Captured images of LDs were processed by binarizing and thinning. Adipocytes were cultured in a chip equipped with the subsystems and the total system was placed in an incubator. The imaging subsystem with a microscope recorded images of LDs of adipose cellular tissue every 1 h. Insulin and adrenaline, which act as accelerators and decelerators of LD size, were used for biochemical stimulation in the experiment. Insulin/adrenaline was supplied through different inlets of the chip depending on the decrease/increase in the ratio between the number of black pixels and white pixels, as defined in the explanation of the imaging subsystem.

Figure 6c shows images captured over 13 h of the experiment. The images show that clusters of LDs were evenly distributed and changed in shape and location in a frame. Feedback control of the size of LDs was performed as shown in the graph in Fig. 6d. Switching between insulin/adrenaline for biochemical stimulation was automatically performed. In most cases, insulin/adrenaline was supplied depending on the decrease/increase in the ratio of white pixels correlating to the size of LDs. The biochemical decelerator was automatically supplied when the ratio of LDs increased over a set value as a threshold, whereas accelerator was automatically supplied when LDs decreased over the threshold. The threshold was set at 0.3% of change in the ratio in the experiment, whereas it could be optionally set to a desired value.

The ratio of LDs decreased despite of supplying insulin at 1 h, 2 h, 6 h, and 11 h. The ratio of LDs increased despite of supplying adrenaline at 9 h. It was likely that these system behaviors were caused by the movement of clusters of LDs out of the frame. The system compared two images of the present frame and the past frame, where the image of when the decrease/increase behavior switched was updated as the past frame for comparison. Algorism updating the past image for comparison by an image of the immediately preceding frame was employed to avoid influence of disturbance in imaging. The algorism successfully worked as programmed in the experiment.

Switching from insulin to adrenaline was carried out by the system when the accelerating effect by insulin was confirmed at 5 h and 8 h. At the same time, switching from adrenaline to insulin was automatically executed at 1 h, 6 h and 11 h. This experiment aimed to verify feedback control by the total system. The system demonstrated automated feedback control by switching in biochemical stimulation and thus achieved the aim in this study. Although there is room for investigation and the further development of system performance, the performance will rise with increasing experiments using the system in future.

Consequently, we anticipate that our PC-controllable cell and tissue system will be a useful technology for exploring feedback control of organs-on-a-chip. The system has potential to contribute to tissue engineering beyond the limitation of human operation due to advantages of PC-controlled automation combining functional subsystems.

## Materials and methods

### C2C12 myotubes

C2C12 myoblasts (American Type Culture Collection, Manassas, VA, USA) were grown in DMEM (4.5 g glucose/L) supplemented with 10% fetal bovine serum (FBS) and 100 U/mL penicillin and 100 μg/mL streptomycin (Nacalai Tesque, Japan)^30,37^. When the myoblasts were approximately 90–100% confluent, the cells were differentiated into myotubes via incubation in DMEM containing 2% horse serum (Thermo Fisher Scientific/Sigma-Aldrich Co. LLC). The medium was changed every 2 days. On day 4 after the differentiation, C2C12 myotubes were analyzed.

### 3T3-L1 adipocytes

3T3-L1 fibroblasts (American Type Culture Collection, Manassas, VA, USA) were cultured in DMEM (1.0 g glucose/L), supplemented with 10% FBS (Cell Culture Bioscience/ Thermo Fisher Scientific K K.), 100 U/mL penicillin, and 100 μg/mL streptomycin (Nacalai Tesque, Japan). To induce differentiation, cells were grown to confluence and maintained for an additional 48 h, and the medium was replaced with DMEM containing 1 μM dexamethasone, 0.5 mM 3-isobutyl-1-methylxanthine (IBMX), and 5 μg/mL insulin for 48 h. The medium was replaced by DMEM containing 5 μg/mL insulin every 2 days^25,37^. On day 12 after the differentiation, 3T3-L1 adipocytes were analyzed.

### Biochemical stimulation

Chemicals were used to stimulate cells in this study. Insulin and adrenaline, which act as accelerators and decelerators of LD size, were used for biochemical stimulation in the experiment. The excess dietary free fatty acids are esterified to inert triglycerides (TG), which are stored in lipid droplets (LDs) in adipose tissue (i.e., lipogenesis), whereas energy demand is increased such as starvation or exercise, TGs are hydrolyzed to free fatty acids and glycerol (i.e., lipolysis)^25^. On day 12 after cell differentiation, lipogenic or lipolytic stimulation was applied to differentiated 3T3-L1 adipocytes as follows. After washing twice with PBS, the cells were incubated with DMEM in the presence of 5 μg/mL insulin or 1 μM adrenaline at 37 °C, as the lipogenic or lipolytic stimulation, respectively^25^.

### Electrical stimulation

SM cells were electrically stimulated to induce their contraction. The conditions were 40 V_pp_ and 40 Hz with 1 s and 4 s as the duration time and the relaxation time, respectively. On day 5 after cell differentiation, C2C12 myotube contraction was induced using an electrical stimulation system, as previously described^38,39^, with minor modifications. Briefly, the cells were washed twice with PBS and were maintained in DMEM containing 2% horse serum. 10 cm cell culture dish was connected to the electrical stimulation apparatus with parallel carbon electrodes spaced 5 cm apart, and were stimulated with a function generator (FGX-295, TEXIO Co.). Differentiated C2C12 myotubes were stimulated with 40 Hz electric pulses at 40 V and for interval pulse durations (1 s of stimulation duration and 4 s of rest intervals) in an incubator maintained at 37 °C to avoid cell damage.

### Metabolite measurement

Acute high-frequency stimulation of myotubes increased glucose uptake, whereas increased cell production of lactate, which reflects muscle contraction to electrical stimuli^39^. Thus, we measured glucose and lactate concentration in the media by an external biosensor (BF-7, Oji Scientific Instruments) which can detect metabolites such as lactate and glucose by introducing the corresponding kit for the objective metabolites. After contraction, the conditioned media were collected at indicated time points, and the samples (40 μL) with phenol red were directly applied to the external biosensor.

### Triglyceride concentration measurement

On day 0, 2, 4, 6, 8, or 10 after induction of differentiation, 100 μL of Lysis Buffer (10 mM Tris, pH 7.4, 1 mM EDTA, 0.1% Triton X-100) was added to the cells of each well (12 well plate), and the cells were recovered. Intracellular triacylglycerol was measured by the TG E-test Wako (FUJIFILM Wako Pure Chemical Corporation, Japan)^37^.

### Statistical analysis

Changes in l-lactic acid variables throughout experimental session between the conditions were analyzed using two-way (condition × time) analysis of variance. Changes in the ratio of LDs, triglyceride concentration, and amount of muscle contraction were analyzed using one-way analysis of variance. Specific differences were identified with a Bonferroni post hoc test. The statistical significance level was defined at *P* < 0.05, and all values were the mean ± standard deviation.

### PC controllable cell and tissue system and experimental setup

The PC controllable cell and tissue system was composed of elemental subsystems: Culture subsystem for culture media exchange and biochemical stimulation, Electrical stimulation subsystem, Imaging subsystem, Sampling & dispensing subsystem in combination with an external analyzer. It is possible to use a commercialized dish and micro-well-plate as well as original microchip as culture environment for our system. 10 cm cell culture dish (GRN 664160-013, Greiner Bio-One) was mainly used in demonstration of this study. The cell and tissue system was controlled by PC (×2 210 G2, Hewlett-Packard Company) in this study. LabVIEW (National Instruments Co.) was used to operate equipment. The culture environment was prepared in CO_2_ Incubator (HERA cell 240i, Thermo Fisher Scientific K.K.). The condition of incubator was kept at 37 °C and 5% CO_2_.

In the culture subsystem and sampling & dispensing subsystem, a power-supply device(S8FS-G10012C, Omron Co.), a terminal block (DIO-1616HN-USB, Contec Co.), a pump (77120-42, Cole-Parmer Instrument Co.), electromagnetically driven valves (LVM102R-6A-10, SMC Co.) were used. Container for dispensed sample was stored in 1.5 ml microtube (131-715CS-N, Watson Co.). Samples of metabolite were analyzed by an external biosensor (BF-7, Oji Scientific Instruments).

In the electrical stimulation subsystem, a function generator (FGX-295, TEXIO Co.) formed electrical stimulation signal based on instruction by LabVIEW on PC. The electrical stimulation signals were applied to cellular tissue by carbon electrode (φ 4.3 mm, C-TASK Co.). Generated signals were measured and confirmed by an oscilloscope (TBS1062, Tektronix, Inc.).

An imaging subsystem employed Invert microscope (CKX41, Olympus Co.) and USB camera (AUSB3-1500K, Arms system Co.). OpenCV (Intel Co.) was used for image processing of acquired images.

## Supplementary Information

Supplementary Figures.

Supplementary Videos.

## Data Availability

All data generated or analyzed during this study are included in this published article.
