# Supplementary material for: Cell and tissue system capable of automated culture, stimulation, and monitor with the aim of feedback control of organs-on-a-chip
Source: Sci Rep. 2021 Feb 4;11:2999. doi: 10.1038/s41598-020-80447-2 (PMC7862322; doi:10.1038/s41598-020-80447-2)
Supplement: Supplementary file 2 — Supplementary Videos. [file 41598_2020_80447_MOESM2_ESM.docx]

**Cell and tissue system capable of automated culture, stimulation, and monitor with the aim of feedback control of organs-on-a-chip**

# Satoshi Konishi^1,2,3*^, Takeshi Hashimoto^3,4^, Tsubasa Nakabuchi^2^, Takatoshi Ozeki^2^, Hiroki Kajita^2^

^1^Department of Mechanical Engineering, College of Science and Engineering, Ritsumeikan University, Kusatsu, 525-8577, Japan

^2^ Graduate Course of Science and Engineering, Ritsumeikan University, Kusatsu, 525-8577, Japan

^3^ Ritsumeikan Global Innovation Research Organization, Ritsumeikan University, Kusatsu, 525-8577, Japan

^4^ College of Sport and Health Science, Ritsumeikan University, Kusatsu, 525-8577, Japan

[^*^konishi@se.ritsumei.ac.jp](mailto:*konishi@se.ritsumei.ac.jp)

**Supplementary Videos**

**Supplementary Video S1. Movie showing muscle contraction.**

**Supplementary Video S2. Image processing for the analysis of magnitude of contraction.**
